# Comparison of palonosetron, granisetron, and ramosetron for the prevention of postoperative nausea and vomiting after laparoscopic gynecologic surgery: a prospective randomized trial

**DOI:** 10.1186/s12871-015-0102-0

**Published:** 2015-09-03

**Authors:** Won-Suk Lee, Kwang-Beom Lee, Soyi Lim, Young Gin Chang

**Affiliations:** 1 Department of Surgery, Gil Medical Center, Gachon University, School of Medicine, Incheon, South Korea; 2 Department of Gynecology, Gil Medical Center, Gachon University, School of Medicine, Incheon, South Korea; 3 Department of Anesthesiology Surgery, Gil Medical Center, Gachon University, School of Medicine, Incheon, South Korea

## Abstract

**Background:**

Selective 5-hydroxytryptamine type 3 (5-HT3) receptor antagonists are reported to have potent antiemetic effects for postoperative nausea and vomiting (PONV). The purpose of this study was to prospectively evaluate the efficacy of palonosetron, granisetron, and ramosetron for the prevention of PONV in patients undergoing laparoscopic gynecologic surgery.

**Methods:**

In this prospective, randomized observational study, 105 healthy female patients who were undergoing laparocopic hystectomy under general anaesthesia were enrolled (clinical trial number: NCT01752374, www.clinicaltrials.gov). Patients were divided into three groups: the palonostron (0.075 mg i.v.; *n* = 35), the granisetron group (3 mg i.v.; *n* = 35), and the ramosetron group (0.3 mg i.v.; *n* = 35). The treatments were given before the end of surgery. The incidence of PONV, severity of nausea/vomiting, and the use of rescue antiemetic requirements during the first 48 h after surgery were evaluated.

**Results:**

The overall incidence of PONV was 33.3 % for this series. The number of complete responders at 48 h after the surgery was 21 (60.0 %) for palonosetron, 24 (68.6 %) for granisetron, and 26 (71.4 %) for ramosetron, representing no statistical difference (*P* = 0.086).

**Conclusions:**

There were no significant differences in the overall incidence of postoperative nausea and vomiting and complete responders for palonosetron, granisetron and ramosetron group.

**Trial registration:**

Clinical trial number: NCT01752374, www.clinicaltrials.gov.

## Background

Laparoscopic surgeries are the second most common cause of postoperative nausea and vomiting (PONV), a frequent and disturbing complication of surgery and anesthesia [1]. The incidence of PONV after gynecological laparoscopy is reported to be nearly 80 % [2] and can result in prolonged hospital stay and recovery times.

Numerous antiemetics have been studied to prevent and treat PONV after laparoscopic abdominal surgery, including antihistamines, anticholinergics and benzamide [3–5]. However, these agents can cause undesirable side effects such as sedation, dry mouth and hypotension.

Selective serotonin 5-hydroxytryptamine type 3 (5-HT_3_) receptor antagonists have a well-established role in the prophylaxis and treatment of PONV due to their efficacy and fewer side effects compared to other antiemetics [6]. Most 5-HT_3_ receptor antagonist research has focused on ondansetron, and the antiemetic efficacy of these compounds has been well established for the prevention and treatment of chemotherapy-induced emesis, as well as for PONV [6, 7].

Granisetron selectively blocks the 5-HT_3_ receptor with a relatively short half-life of 4 to 9 h. Ramosetron is a recently developed selective 5-HT_3_ receptor antagonist, which exhibits a significantly greater binding affinity for 5-HT_3_ receptors and a slower dissociation rate compared to older 5-HT_3_ receptor antagonists, resulting in more potent and longer effects. Palonosetron is a second-generation 5-HT_3_ receptor antagonist with an even higher receptor binding affinity, and a prolonged mean half-life of about 40 h [8]. We hypothesized that long acting palonosetron treatment would be more effective in lowering the incidence of PONV, compared to treatment with granisetron and ramosetron. The purpose of this study was to prospectively evaluate the efficacy of palonosetron, granisetron, and ramosetron in the prevention of PONV in patients undergoing laparoscopic gynecologic surgery.

## Methods

### Inclusion criteria

**Fig. 1:**
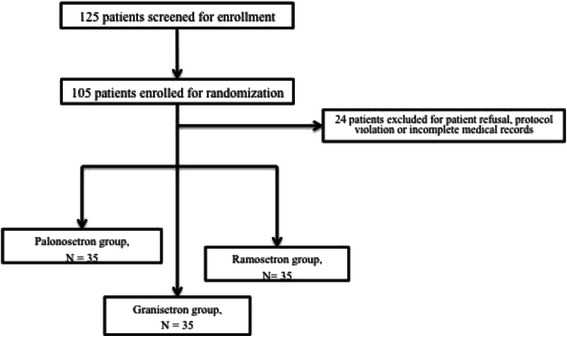
Patient allocation

The study proposal was finalized and approved by Gachon University, Gil Hospital institutional review board before the investigation was initiated. Written informed consent was acquired from all participants before enrollment in the study. This investigator-initiated, prospective, non-blinded, randomized controlled trial was performed at Gachon University, Gil Hospital between November 2011 and June 2013. Using a random number table, patients were randomly assigned to receive palonosetron (*n* = 35), ramosetron (*n* = 35), or granisetron (*n* = 35; Fig. 1).

### Exclusion criteria

Exclusion criteria were as follows: Allergy to any of the experimental drugs, opioid dependence, a history of PONV and motion sickness, use of antiemetic medication within 24 h prior to surgery, pregnancy, inability to use the patient controlled analgesia (PCA) IV device or to comprehend the 10 cm visual analogue scales (VAS; 0, none; 10, maximum) for pain and nausea assessment, and unwillingness to be enrolled in the study.

### Study protocol

All patients received intramuscular midazolam (0.05 mg/kg) and glycopyrrolate (0.2 mg) as premedication 1 h before anesthesia induction. In the operating room, the vital signs of the patient were continuously monitored using electrocardiogram, pulse oximetry, and measurement of noninvasive arterial pressure. Anesthesia was induced and maintained with propofol (target effect site concentration 2.5–3.5 μg/ml) and remifentanil (target effect site concentration 2.5–5.0 ng/ml) using a target controlled infusion (TCI) pump (Orchestra®, Fresenius Vial, Brezins, France). Rocuronium (0.6 mg/kg) was administered to facilitate tracheal intubation. Anesthesia was maintained with sevoflurane (0.5 ─5 %) and nitric oxide (50 %) throughout surgery.

All patients were ventilated using an S/5 Avance anesthetic machine (GE Healthcare, Madison, WI). After anesthesia induction, patients were mechanically ventilated with constant flow and I:E ratio of 1:2, and tidal volume (TV) set at 8 ml/kg of ideal body weight. Respiratory rate was adjusted to 8–20 breaths/min to maintain end-tidal carbon dioxide concentration (ETCO_2_) of 30–40 mmHg at 60 % inspired oxygen with air [9].

Pneumoperitoneum was established with a closed Veress needle technique, and the intra-abdominal pressure was maintained at 12–14 mmHg. After CO_2_ insufflation, patients were placed in the reverse Trendelenburg position at 20°. Laparoscopic gynecologic surgery was performed through two ports of 10 mm and two ports of 5 mm in the standard position with the legs closed [9].

Patients received a single dose of palonosetron 0.075 mg, granisetron 3 mg, or ramosetron 0.3 mg intravenously (IV) at the end of the surgery, prior to extubation. The experimental medications were prepared in identical 5 cc syringes and administered according to protocol by a nurse. For postoperative pain control, diclofenac 75 mg was administered intramuscularly (IM) at the start of wound closure, and repeated upon patient request. The incidence of nausea and vomiting was recorded during the assessment periods by ward nursing staff not blinded to the treatment drugs.

Nausea was defined as a subjectively unpleasant sensation associated with the urge to vomit. Incidences of vomiting included retching (defined as the labored, spastic, rhythmic contraction of the respiratory muscles without expulsion of the gastric contents) and actual vomiting (defined as the forceful expulsion of gastric contents from the mouth). A complete response to palonosetron, granisetron, and ramosetron was defined as an absence of PONV and no need for further rescue antiemetic drugs. A rescue antiemetic (10 mg metoclopramide) was administered IV upon patient request, if two or more episodes of PONV occurred during the study period, if nausea intensity increased from moderate to severe (VAS > 5) and on vomiting. The primary outcome was incidence of complete responders during the study period. Details of any other adverse effects such as headache and dizziness were also collected. All data were collected by a dedicated research nurse at 6, 24, and 48 h after surgery.

### Statistical analyses

Sample size was calculated using two proportions power analysis on the basis of the primary outcome measure. It was estimated that 30 patients per group would be required for the power analysis (for a power of 80 % and a type 1 error of 5 %) to demonstrate a relative reduction of 25 % in complete response in each group 48 h after surgery. This calculation was based on previously published studies [10, 11]. Student’s *t*-test was used to compare the inter-group differences, and a chi-square test was used for categorical variables. *P*-values were corrected using the Bonferroni method. Values are expressed as counts or the mean ± standard deviation. *P*-values < 0.05 were considered statistically significant. All patients underwent laparoscopic hysterectomy under general anesthesia.

## Results

All patients completed the study. Patient characteristics and basic operative data are presented in Table 1. A total of 125 patients were screened to assess their eligibility for this trial (Fig. 1) and 105 patients were enrolled in the study. The groups were comparable with respect to age, weight, duration of surgery, and ASA score. The overall incidence of PONV was 19.3 %. The incidence of nausea was highest during the first 6 h (total incidence of 18.1 %) and decreased throughout the study period.

The number of complete responders at 48 h after the surgery was 21 (60.0 %) for palonosetron, 24 (68.6 %) for granisetron, and 26 (74.3 %) for ramosetron. The differences between the groups were not statistically significant (*P* = 0.086, Table 2). All three groups were comparable across the time intervals examined.

**Table 1:** Patient characteristics (*n* = 105)

	Palonosetron group, *n* = 35	Granisetron group, *n* = 35	Ramosetron group, *n* = 35	*P*-value
Age, year	51.5 ± 16.3	52.5 ± 15.7	48.2 ± 12.1	0.42
Height (cm)	155.3 ± 3.1	157.1 ± 6.1	157.2 ± 3.3	0.65
Weight (kg)	60.1 ± 4.9	59.3 ± 5.1	56.3 ± 4.3	0.55
Operation Time(min)	100.2 ± 34.1	99.4 ± 25.3	92.6 ± 24.9	0.46
Anesthesia Time(min)	128.1 ± 47.5	123.5 ± 35.1	120.1 ± 23.0	0.63
Fluid Admin(mL)	522.1 ± 61.2	644 ± 33.3	538 ± 53.9	0.75
ASA class I/II	23/12	24/11	26/9	0.67
Comorbid dis.				0.56
Hypertension	7	8	8	
Diabetes	3	3	4	
Other^a^	1	0	1	

^a^Other comorbid diseases include: Fatty liver, Rheumatoid arthritis

**Table 2:** Number of nausea and vomiting over time in the three study groups (*n* = 105)

	Palonosetron group, *n* = 35 (%)	Granisetron group, *n* = 35 (%)	Ramosetron group, *n* = 35 (%)	*P*-value
0-6 h				
Nausea	6 (17.1)	5 (14.2)	6 (17.1)	0.87
Vomiting	1 (2.9)	0 (0)	1 (2.9)	N/A
Rescue drug	2 (5.7)	1 (2.9)	1 (2.9)	0.69
6-24 h				
Nausea	3 (8.6)	4 (11.4)	4 (11.4)	0.73
Vomiting	1 (2.9)	0 (0)	0 (0)	N/A
Rescue drug	1 (2.9)	0 (0)	0 (0)	N/A
24-48 h				
Nausea	2 (5.7)	1(2.9)	1 (2.9)	0.43
Vomiting	0 (0)	0 (0)	0 (0)	N/A
Rescue drug	0 (0)	0 (0)	0 (0)	N/A
No. complete responder	21, 60.0 %	24, 68.6 %	26,74.3 %	0.086

N/A; not applicable due to small number of samples

The number of subjects in each of the three study groups experiencing at least one episode of vomiting within the three time intervals is shown in Table 2. More subjects suffered vomiting in the early phase (0–6 h) of postoperative period in the palonosetron and ramosetron groups compared with the granisetron group, which had no vomiting during this period. However, this difference was not statistically significant.

The incidence of most of the common adverse events, such as headache and dizziness, was similar among the three groups, and no clinically significant treatment-related adverse events were observed.

## Discussion

In the present study, all three antiemetics were shown to be equally effective in preventing nausea and vomiting. There were no statistically significant differences in the number of complete responses between the three groups. A variety of 5-HT_3_ antagonists have been used to manage PONV [12]. It is generally accepted that all 5-HT_3_ antagonists have a similar mechanism of action (selective or competitive binding to 5-HT_3_ receptors) as well as comparable efficacy and safety profiles [5, 10]. This is the first direct comparison between these three antiemetics for their role in case of laparoscopic gynecologic surgery.

The reported incidence of PONV is 30 to 80 % within the first 24 h after laparoscopic surgery when no prophylactic antiemetic is administered [5, 3, 13]. The present study reports an overall incidence of 33.3 % within the first 48 h post-surgery, without any statistical difference between the three groups. The high incidence during the first 24 h of surgery may be explained by the central action of carbon dioxide (CO_2_), stretching of the peritoneum and diaphragm, and increased blood pressure in the peritoneal cavity after CO_2_ insufflation during laparoscopic surgery, as previously reported by Ryu et al. [3]. All these factors are considered to provoke nausea and vomiting by reducing the blood flow and releasing emetogenic substances, including serotonin [14, 15].

The reported efficacy of ramosetron for the prevention of chemotherapy-induced emesis is similar to that of granisetron [16]. However, ramosetron appears to have a longer duration of action; its effects last for 24 h after cisplatin-based chemotherapy [17]. Pal et al. [18] found that ramosetron is also more effective than palonosetron and ondansetron during the early postoperative period. However, we did not find any significant difference between patients treated with these compounds in the present study. Of note, we used remifentanil to achieve the desired analgesic effect. Remifentanil infusion during desflurane- or propofol-induced anesthesia facilitates early recovery without immensely increasing PONV, pain or the need for rescue medication after laparoscopic surgery [19].

We are unaware of any published study simultaneously evaluating the efficacy of palonosetron, granisetron, and ramosetron. Discrepancies exist between studies examining the effects of each 5-HT_3_ antagonist individually [20]. To the best of our knowledge, ours is the first study to evaluate the efficacy of palonosetron, granisetron, and ramosetron as prophylactics for preventing PONV in patients undergoing uniform laparoscopic gynecologic surgery. Aspinall and Goodman suggested that if active drugs are available, placebo-controlled trials are unethical because PONV is a very disturbing and distressing event that can occur after laparoscopic surgery [20]. In the current study, three commercially available 5-HT_3_ antagonists were used as antiemetics to prevent PONV after laparoscopic surgery. We found that the three agents yielded similar numbers of complete responses (i.e., no postoperative nausea or vomiting and no rescue antiemetic administration required) between 0 and 48 h after surgery.

We observed relatively similar numbers of complete responders in each group, in contrast with previously published reports (Table 3). The most frequently reported adverse events of 5-HT_3_ receptor antagonists are dizziness and headache [6]. The adverse events observed in our study were similar among all three groups of patients.

**Table 3:** Results of studies evaluating outcomes of PONV patients undergoing laparoscopic surgery

Authors	Prospective study	Number of patients enrolled	Year of study	Type of PONV	Complete responder
					Incidence, (%)
Yun et al.	No	98	2010	Az, On	35-51
Ryu et al.	No	120	2010	On, Ra	23-40
Fujii et al.	Yes	120	1999	Ra, Gr	87-90
Swaika et al.	Yes	87	2011	Ra, Pa	38-66
Current study	Yes	105	2012	Pa, Ra, On	60-71

*Az* azasetron; *On* ondansetron; *Pa* palonostron; *Ra* ramosetron; *Gr* graniestron

The major limitations of the current study are: (1) We compared the efficacy of palonosetron, ramosetron, and granisetron at their known optimal doses, not at equipotent doses, and (2) this was not a double-blinded study. We did not administer equipotent doses of the compounds as they were unknown at the time of commencement of the study. Investigations on a larger scale are needed to assess the equipotency of palonosetron, ramosetron, and granisetron.

## Conclusions

In conclusion, our study demonstrated that palonosetron, ramosetron, and granisetron are equally effective as antiemetics against postoperative nausea and vomiting in patients undergoing laparoscopic gynecologic surgery.
